# Impact of organic manure on fruit set, fruit retention, yield, and nutritional status in pomegranate (*Punica granatum* L. “Wonderful”) under water and mineral fertilization deficits

**DOI:** 10.7717/peerj.10979

**Published:** 2021-03-03

**Authors:** Mahmoud Abdel-Sattar, Khalid F. Almutairi, Abdulwahed M. Aboukarima, Mohamed El-Mahrouky

**Affiliations:** 1 Department of Plant Production/ College of Food and Agriculture Sciences, King Saud University, Riyadh, Riyadh, Saudi Arabia; 2 Pomology Department, Faculty of Agriculture, Alexandria University, Alexandria, Alexandria, Egypt; 3 Agricultural Engineering Research Institute, Agricultural Research Center, Dokki, Giza, Egypt; 4 Department of Agricultural Engineering/ College of Food and Agriculture Sciences, King Saud University, Riyadh, Riyadh, Saudi Arabia; 5 Soil Science Department/ College of Food and Agriculture Sciences, King Saud University, Riyadh, Riyadh, Saudi Arabia

**Keywords:** Deficit irrigation, Mineral fertilizer, Organic manure, Pomegranate

## Abstract

This research was conducted on mature pomegranate (*Punica granatum* L. “Wonderful”) trees growing at a site located in North Coast, Matrouh Governorate, Egypt. The aim was to investigate the impacts of different irrigation regimes in combination with different fertilizer regimes on the fruit set, fruit retention, yield, and nutritional status of the trees. The experimental factors were arranged in a split-plot design, with four replicates per treatment combination. The results indicated that all of the characteristics measured, including leaves nutritional status, percentages of fruit set, fruit drop, fruit retention, fruit cracking, fruit sunburn, and marketable fruit, and yield were significantly affected by the interaction between the irrigation treatment which denoted by percentages of reference evapotranspiration (ETo) and fertilizer regime. The application of 75% mineral fertilizer + 25% organic manure under deficit irrigation of 80% ETo increased the yield by an average of 18.23% over the 2 years compared with 100% mineral fertilization under full irrigation, while 50% mineral fertilizer + 50% organic matter under 80% ETo gave the maximum percentage of marketable fruit (86.23% and 86.84% in 2018 and 2019, respectively). The maximum water use efficiency was obtained with the 80% ETo treatment combined with 75% mineral fertilizer + 25% organic manure in both seasons with values of 9.69 and 10.06 kg/m^3^ applied water, respectively. These results demonstrate that under the field conditions at the experimental site, the fruit set and retention could be improved by applying a reduced amount of mineral fertilizer in combination with organic manure and less irrigation water.

## Introduction

Fruits are adding value of earth’s diversity and fundamental to all life. They include high content of non-nutritive, nutritive, and bioactive compounds such as flavonoids, phenolics, anthocyanins, phenolic acids, as well as nutritive compounds such as sugars, essential oils, carotenoids, vitamins, and minerals. With their rich nutrients, fantastic flavor and taste, excellent medicinal value and health care functions, wild edible fruits have been continuously well received by consumers as important economic crops (Ercisli & Esitken, 2004; Eyduran et al., 2015; Colak et al., 2019; Senica, Stampar & Mikulic-Petkovsek, 2019).

The pomegranate (*Punica granatum* L., Punicaceae) has been cultivated for 5,000 years and is grown successfully in tropical and sub-tropical regions. It is a well-known tree in the central Asian region, Morocco, Spain, and Egypt, where it is valued for its antioxidant and nutritional characteristics (Parvizi & Sepaskhah, 2015). There are several popular cultivars of pomegranate, among which Wonderful is one of the most widely used for the production of commercial juice, which has health benefits as summarized in Hmid et al. (2018) and the arils are consumed as fresh fruit.

The pomegranate can be grown in arid and semi-arid regions under deficit or full irrigation conditions (Tavousi et al., 2015). The production of pomegranate depends on both the irrigation regime and fertilization practices used in different soil types (Taha, 2018). Most recent research studies on pomegranates have dedicated on fruit features (Lazare et al., 2020). However, the scientific base for the agronomic desires of the pomegranate is still ambiguous, and practiced empirical recommendations are inadequate (Lazare et al., 2020). Thus, there is a necessity for sustainable farming in which soil fertility is sustained by the use of low-cost renewable resources that are freely obtainable on the farm (Baghdadi et al., 2018). In many parts of the world, fruit trees require an efficient water supply but there has been a rapid reduction in water availability. Consequently, new farm practices are needed in pomegranate orchards to overcome the limited water supply (Kang et al., 2002). Fortunately, pomegranate trees are very tolerant of drought and severe water stress (Pourghayoumi et al., 2017), but adequate soil moisture around the tree improves plant vigor and fruit yield. Therefore, it is essential for farm practices that allow pomegranate cultivation under water deficit conditions fertilization to be identified and introduced.

The management of soil fertility and the use of organic fertilizers as alternatives to chemical fertilizers are the main concerns in agricultural production (El-Shazly et al., 2015). The main reason contributing to lessening crop yields is reduced soil fertility produced by continuous cropping without the addition of adequate manure and mineral fertilizers (Ndayisaba, 2013). The application of organic manure affects not only the nutrient supply to the plants but also the chemical and physical characteristics of the soil (EL-Kouny, EL-Assar & Mohamed, 2004; Abbas et al., 2006). However, the combined application of organic and chemical sources, which is usually stated to as integrated nutrient management, is usually familiar as a way of sustainably increasing crop productivity (Mahajan, Bhagat & Gupta, 2008). Gonzalez, Alvarez & Matheus (2001) reported that increased plant height in response to applications of combined fertilizer (chemical fertilizer and organic manure) is attributed to more availability of N from both urea and manure throughout the growing season.

Tirol-Padre et al. (2007) reported that the organic manure may be beneficial to both the soil and crop in the long term. Moreover, Kanal & Kuldkepp (1993), Mottaghian et al. (2008) and Aisueni et al. (2009) showed that, generally, the organic manure used in combination with mineral fertilizers to improve crop growth and yield in the short term, although Kassem & Marzouk (2002) and Marzouk & Kassem (2011) showed that organic manure alone improved the fruit quality, nutritional value, and yield of Flame Seedless grapes (*Vitis vinifera* L.) and Zaghloul dates (*Phoenix dactylifera* L.), respectively. Moreover, organic manure uses improved soil organic matter and soil nutrients, which were released slowly and steadily and were efficiently used during later growth stages of crop (Baghdadi et al., 2018).

Nutrient fertilization are significantly influences fruit production as the plant nutritional status affects flowering, vegetative growth, fruit retention, fruit set and has a noticeable influence on yield and fruit quality (Hunter et al., 2011; Srivastava & Malhotra, 2017; Lyu et al., 2019; Davarpanah et al., 2020). Moreover, the use of chemical fertilizer rather than organic manure has some significance because the former readily deliveries nutrients to crops, which helps rise both growth and yield (Baghdadi et al., 2018). Among the numerous reasons responsible for increasing crop production, the use of balanced fertilizer at the right quantity, right time, source and scheme shows a vital role in improving productivity (Tanari et al., 2019). Therefore, the aim of the present study was to investigate the effects of combining deficit irrigation practices with different mixtures of organic manure and mineral fertilizers on fruit set, fruit retention, yield, and nutritional status in Wonderful pomegranate to improve our understanding of pomegranate production under deficit irrigation and mineral fertilization conditions.

## Materials & Methods

### Experimental site and plant materials

This study was carried out over two continuous growing seasons (2018 and 2019) using 6-year-old pomegranate trees of the Wonderful cultivar planted in a private orchard in North Cost, Matrouh Governorate, Egypt. The trees were planted at a spacing of 2.5 × 4 m and supplied with water via a drip irrigation system that had two lines per tree and four drippers (8 L/h) per line. Before conducting the experiment, three soil samples were collected at a depth at 0–90 cm for analysis of various physicochemical properties, as shown in Tables 1 and 2. The soil texture in the experimental site is shown in Table 2.

Mineral fertilizer (100%) containing 240 units of N, 60 units of P_2_O_5_, 192 units of K_2_O, 71.65 units of CaO, and 50 units of MgO was applied with the irrigation water weekly from March 1 through to mid-September in both growing seasons. Organic manure (100%) containing 0.24 units of N, 0.07 units of P_2_O_5_, 0.27 units of K_2_O, 0.13 units of CaO, and 0.07 units of MgO was added at a rate of 19.35 kg/tree in November of both years. Organic manure analysis was performed using the standard procedures described by Bertran & Andrease (1994) and showed that the organic fertilizer applied in this study contained 44% organic matter, 22% organic carbon, 1.24% N, 0.38% P_2_O_5_, 1.40% K_2_O, 0.68% CaO, 0.36% MgO, 4500 ppm Fe, 450 ppm Mn, 125 ppm Zn, and 44 ppm Cu and had a moisture content of 11.4 d.b (dry basis) and a C/N ratio of 11:1 (values are averages of both growing seasons).

**Table 1:** Soluble cations and anions in the soil at the study site.

Soil depth (cm)	Soluble cations (Meq/L)				Soluble anions (Meq/L)		
	Na^+^	Ca^2+^	Mg^2+^	K^+^	HCO${}_{3}^{-}$	Cl^−^	SO${}_{4}^{2-}$
0–30	4.2	2.8	2.4	1.0	2.2	5.4	2.8
30–60	4.3	3.1	2.6	0.8	2.1	5.8	2.9
60–90	4.5	3.6	2.5	0.7	2.0	6.0	3.3

**Table 2:** Physical and chemical properties of the soil at the study site.

Soil depth (cm)	Sand (%)	Clay (%)	Silt (%)	Soil texture	pH	Electric conductivity (dS/m)	CaCO_3_ (%)	Organic matter (%)
0–30	43.10	13.13	43.77	Loam	8.1	1.04	29.75	0.32
30–60	26.60	22.17	51.23	Silt loam	7.8	1.08	18.95	0.28
60–90	19.40	20.39	60.21	Silt loam	7.8	1.13	12.14	0.17

All pomegranate trees were cultivated using the normal agricultural practices applied in this pomegranate orchards which follow the recommendations of the Ministry of Agriculture, Egypt. The experimental site included a total of 240 trees, and a split-plot design was used in both growing seasons, according to Snedecor & Cochran (1990). The main plots included three levels of irrigation treatments: full irrigation equivalent to 100% of the reference evapotranspiration rate (100% ETo) as a control (I1), 80% ETo (I2), and 60% ETo (I3). The sub-plots included five fertilizer regimes: 100% mineral fertilizer (T1), 100% organic manure (T2), 75% mineral fertilizer + 25% organic manure (T3), 50% mineral fertilizer + 50% organic manure (T4), and 25% mineral fertilizer + 75% organic manure (T5). There were four replications per treatment combination and four trees per replicate.

The necessary climatic data for estimating the daily ETo were collected from the nearest weather station, and the ETo (mm/day) was calculated according to the FAO 56 Penman–Monteith equation (Allen et al., 1998):

(1) \begin{eqnarray*}{\mathrm{ET}}_{\mathrm{O}}= \frac{0.408\Delta \left( \mathrm{Rn}-\mathrm{G} \right) +\gamma \left( \frac{900}{\mathrm{T}+273} \right) {\mathrm{U}}_{2} \left( {\mathrm{e}}_{\mathrm{s}}-{\mathrm{e}}_{\mathrm{a}} \right) }{\Delta +\gamma \left( 1+0.34{\mathrm{U}}_{2} \right) } \end{eqnarray*}

where Δ is the slope of the saturated vapor pressure/temperature curve (kPa/°C), *γ* is the psychometric constant (kPa/°C), U_2_ is the wind velocity at 2 m height (m/s), Rn is the total net radiation at the crop surface (MJ/m^2^/day), G is the density of the soil heat flux (MJ/m^2^/day), T is the mean daily air temperature at 2 m height (°C), e_s_ is the saturation vapor pressure (kPa), and e_a_ is the actual vapor pressure (kPa).

Crop evapotranspiration (ETc, mm/day) was then determined as follows:

(2) \begin{eqnarray*}ETc=Kc\times ETo\end{eqnarray*}

where Kc (dimensionless) is the crop coefficient, which increased from an initial value of 0.32 in March to a peak value of 0.74 in July, August, and September, according to Intrigliolo et al. (2011). Deficit irrigation was tested at 80% ETo and 60% ETo. The amount of irrigation water applied (L/tree) and the number of irrigations with each irrigation regime during the two growing seasons are shown in Table 3. During the period from January to December seasons 2018 and 2019 under the field experiment, the rainfall was with an annual mean 157.2 and 168.9 mm, respectively. The data were obtained from weather station near the experimental site (Table 3).

**Table 3:** Amount of precipitation and irrigation water applied with each irrigation treatment during the two growing seasons.

Month	Precipitation (mm/season)		No. Irrigations	Total volume of irrigation water applied (L/tree/season)					
	2018	2019		2018			2019		
				I_1_	I_2_	I_3_	I_1_	I_2_	I_3_
January	36.4	54.8	2	100	80	60	97	78	58
February	5.3	3.6	4	200	160	120	115	92	69
March	3.8	1.3	8	480	384	288	458	366	275
April	0.1	0.2	8	560	448	336	535	428	321
May	0	0	12	840	672	504	798	638	479
June	0	0	12	840	672	504	798	638	479
July	0	0	12	840	672	504	790	632	474
August	0	0	12	840	672	504	794	635	476
September	1.0	0.8	8	560	448	336	532	426	319
October	2.3	5.9	4	240	192	144	229	183	137
November	49.0	45.9	2	120	96	72	115	92	69
December	59.3	56.4	2	100	80	60	97	78	58

### Measurement of nutritional status

A total of 30 leaves were collected manually from non-fruiting shoots in the middle part of each tree in the first week of August each year. The nutritional status of the pomegranate trees was estimated by determining the leaf mineral constituents and total leaf chlorophyll content. The total leaf chlorophyll content was determined using fresh leaf samples, according to the method described by Yadava (1986), using the Chlorophyll Meter SPAD-502 (Minolta Camera Co., Ltd., Japan). The SPAD-502 measurements were conducted on fresh leaf samples and the adaxial side of the leaves was always placed toward the emitting window of the instrument and major veins were avoided.

To determine the leaf mineral contents, the leaves were washed with tap water and distilled water and then dried at 65−70 °C for 72 h (Reisenauer, 1976). A 1-g sample of dried ground leaf material from each tree was then digested with sulfuric acid and hydrogen peroxide according to Evenhuis & De Waard (1980). The nitrogen (N) content (%) of the digested solution was determined by the micro-Kjeldahl method following the methods described by Chapman & Pratt (1961), and total N and P were colorimetrically determined according to the methods described by Evenhuis (1976) & Murphy & Riley (1962), respectively. K was determined by flame photometry, as described by Jackson (1967), and the Ca and Mg contents (%) were determined using an atomic absorption spectrophotometer (model 305B) according to Jones (1977).

### Percentages of fruit set, drop, and retention

At the time of flowering (April in both years), two main branches growing in different directions were selected and tagged on each tree and the percentages of fruit set (FSP), fruit drop (FDP), and fruit retention (FRP) were determined as follows:

(3) \begin{eqnarray*}& \mathrm{FSP}= \frac{\text{TNDF}}{\text{TNPF}} \times 100\end{eqnarray*}

(4) \begin{eqnarray*}& \mathrm{FDP}= \frac{\text{TNFS-TNFH}}{\text{TNFS}} \times 100\end{eqnarray*}

(5) \begin{eqnarray*}& \mathrm{FRP}= \frac{\text{TNFH}}{\text{TNFS}} \times 100\end{eqnarray*}

where TNDF is the total number of developed fruitlets, TNPF is the total number of perfect flowers, TNFS is the total number of fruit set, and TNFH is the total number of fruit at harvest.

### Tree yield and percentages of fruit sunburn, fruit cracking, and marketable fruit

Trees were harvested manually in the second week of October in both years when the fruits reached the ripening stage and became fully collared. The number of sunburned and cracked fruit were determined for each treatment and the percentages of fruit sunburn (FBP), fruit cracking (FCP), and marketable fruit (MFP) were determined as follows:

(6) \begin{eqnarray*}& \mathrm{FBP}= \frac{\mathrm{NBF}}{\mathrm{TNF}} \times 100\end{eqnarray*}

(7) \begin{eqnarray*}& \mathrm{FCP}= \frac{\mathrm{NCF}}{\mathrm{TNF}} \times 100\end{eqnarray*}

(8) \begin{eqnarray*}& \mathrm{MFP}= \left( \frac{\text{TNF-NCF}-\mathrm{NBF}}{\mathrm{TNF}} \right) \times 100\end{eqnarray*}

where NBF is the number of sunburned fruit, TNF is the total number of fruit, and NCF is the number of cracked fruit.

At the time of harvest, five fruit from each tree were collected and weighed, and the average weight was calculated. In addition, the total number of fruit was counted and the average total yield per tree (kg/tree) was calculated.

### Irrigation water use efficiency

The irrigation water use efficiency (kg/m^3^) was determined by dividing the crop yield (kg/ha) by the total amount of irrigation water applied (m^3^/ha), as reported by Zhang (2003).

### Statistical analysis

The data were analyzed using analysis of variance (ANOVA), as reported by Gomez & Gomez (1984), and the means of the various treatments were compared using the least significant difference (LSD) test. All analyses were performed in SAS version 9.13 (SAS Institute, Cary, NC, USA) using the 5% level of significance.

## Results and Discussion

### Effects of the irrigation and fertilizer regimes on nutritional status

**Figure 1:**
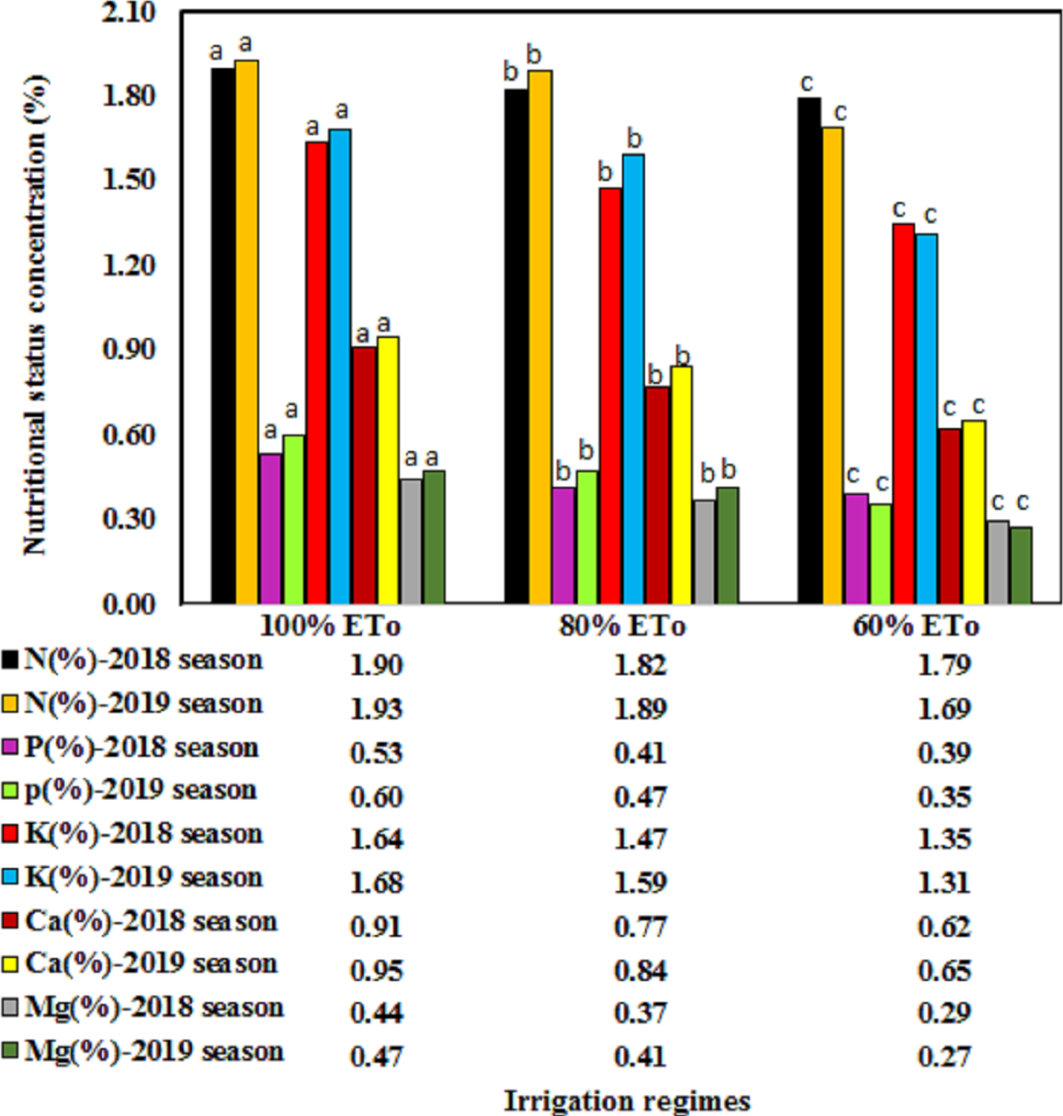
The nutritional status of the Wonderful pomegranate leaves in term of N, P, K, Ca, and Mg content as influenced by irrigation regimes in two seasons.

**Figure 2:**
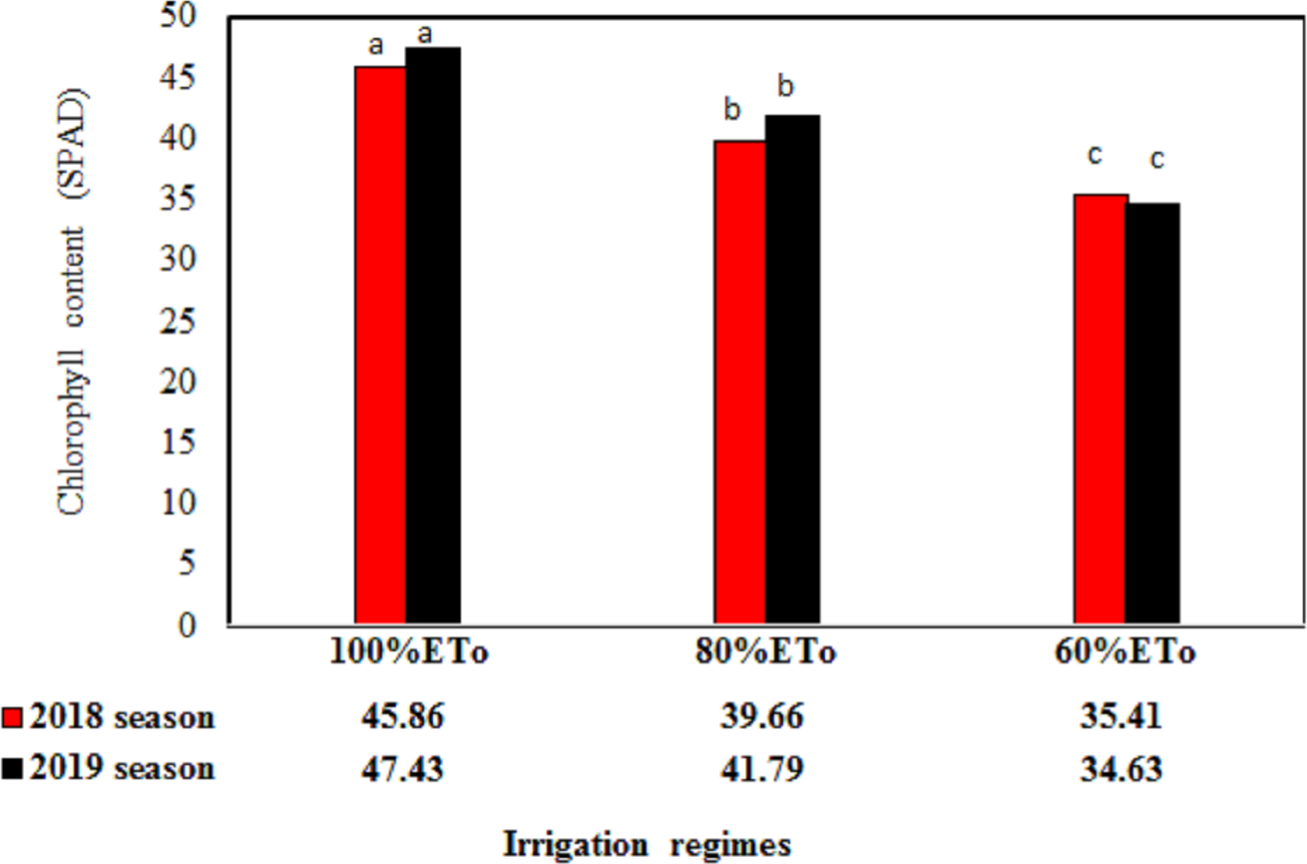
The nutritional status of the Wonderful pomegranate leaves in term of the chlorophyll content as influenced by irrigation regimes in two seasons.

The nutritional status of the Wonderful pomegranate leaves in terms of the P, N, K, Ca and Mg were significantly affected by the irrigation regimes (*P* < 0.05) in both seasons (Fig. 1). Moreover, chlorophyll content was significantly affected by the irrigation regimes (*P* < 0.05) in both seasons (Fig. 2). All of these parameters were fluctuated in both growing seasons and tended to significantly increase with an increasing irrigation level. These findings support those obtained by Khattab et al. (2011), who also showed that the P, N, K, and chlorophyll contents of pomegranate leaves could be significantly increased by increasing the amount of irrigation water applied. This effect may result from an increased accessibility of such nutrients under excess soil moisture conditions, which would improve the uptake rate by a tree and also increase the amount of photosynthesis, thereby increasing the leaf area (El-Kassas, 1983).

Reducing the irrigation rate from 5,720 L/tree (100%ETo, control) to 4,576 L/tree (80%ETo) and 3,432 L/tree (60%ETo) resulted in moderate P, N, Ca, K, Mg, and chlorophyll contents in 2018 and will have had benefits for the environment, as Ren et al. (2014) argued that the huge amounts of water applied by farmers could cause many environmental issues wherever the leaching of fertilizer away from the root zone and into groundwater could occur. Furthermore, under treatment 80%ETo, the pomegranate trees had moderate nutritional values during both seasons, suggesting an absence of limiting factors for ETc to be satisfied (Rodríguez et al., 2012; Galindo et al., 2013; Galindo et al., 2014).

All nutritional parameters of P, N, Ca, K, Mg, and chlorophyll contents were significantly affected by the fertilizer regime (*P* < 0.05), with obvious differences between the two growing seasons (Table 4). In both seasons, integrated uses of fertilizer sources of the 75% mineral fertilizer + 25% organic manure regime gave the greatest improvement in nutritional status, with higher percentages of N, K, P, Mg, and chlorophyll than 100% mineral fertilizer or 100% organic manure. Furthermore, lower levels of mineral fertilizer in combination with organic manure (i.e., 50% mineral fertilizer + 50% organic manure or 25% mineral fertilizer + 75% organic manure) also gave reasonable values for the percentage contents of N, K, P, Mg, and chlorophyll compared with 100% mineral fertilizer or 100% organic manure. This may be because each fertilizer contained different essential elements or due to the fact that the organic fertilizer helped to facilitate the accessibility and uptake of most nutrients to the trees (El-Shazly et al., 2015). The addition of organic manure can also enhance soil texture by aggregating the soil particles through the use of various organic molecules, such as polysaccharides, and increasing the activities of microorganisms in the soil, which enhances the biochemical cycling, resulting in an increased availability of elements (El-Shazly et al., 2015). Furthermore, organic manure also improves the physical characteristics of soil by increasing the nutrient and water holding capacity, total pore space, aggregate stability, erosion resistance, and temperature insulation and decreasing the apparent soil density (Shiralipour, McConnell & Smith, 1992). Moreover, integrated uses of fertilizer sources help maintain the fertility status of the soil (Baghdadi et al., 2018).

**Table 4:** Main effects of the five fertilizer regimes on the N, P, K, Ca, Mg, and chlorophyll contents of pomegranate (*Punica granatum* “Wonderful”) leaves in the 2018 and 2019 seasons. Mean values within a column for each season that are followed by different letters are significantly different at *P* ≤ 0.05.

Season	Fertilizer regime	N (%)	P (%)	K (%)	Ca (%)	Mg (%)	Chlorophyll (SPAD)
2018	T_1_	1.83c	0.43c	1.47c	0.70e	0.36c	39.48c
	T_2_	1.70e	0.37e	1.39e	0.84a	0.32e	36.51d
	T_3_	1.95a	0.52a	1.57a	0.72d	0.42a	44.24a
	T_4_	1.90b	0.47b	1.53b	0.77c	0.39b	41.75b
	T_5_	1.80d	0.41d	1.46d	0.81b	0.35d	39.55c
	LSD	0.01	0.01	0.01	0.01	0.001	0.27
2019	T_1_	1.83c	0.46c	1.52c	0.74e	0.37c	40.87d
	T_2_	1.69e	0.40e	1.43e	0.90a	0.33e	38.38b
	T_3_	1.96a	0.55a	1.61a	0.78d	0.44a	46.38a
	T_4_	1.90b	0.51b	1.57b	0.82c	0.41b	40.47b
	T_5_	1.81d	0.45d	1.51d	0.86b	0.36d	40.33b
	LSD	0.02	0.01	0.01	0.01	0.01	3.82

### Effects of the irrigation and fertilizer regimes on the percentages of fruit set, drop, retention, sunburn, and cracking, the percentage of marketable fruit, and yield

Irrigation is known to modify processes related to fruit trees (Martínez-Nicolás et al., 2019). However, in the present study, there were no consistent trends in the percentages of fruit cracking, fruit set, fruit sunburn, fruit drop, fruit retention, or marketable fruit or the yield of Wonderful pomegranate trees due to the different irrigation regimes in either season.

The availability of water for agricultural use is the main challenge for optimal fruit tree cultivation under Mediterranean conditions (Martínez-Nicolás et al., 2019). Lower percentages of fruit cracking, fruit sunburn, and fruit drop can be considered advantageous and were observed under an irrigation deficit, with values of 7.66% and 7.27% fruit cracking under 80%ETo, 7.35% and 7.05% fruit sunburn under 60%ETo, and 22.35% and 23.33% fruit drop under 80%ETo in the 2018 and 2019 seasons, respectively. By contrast, the 100%ETo treatment increased the percentage of fruit cracking. Furthermore, the 80%ETo treatment also increased the percentages of fruit set, fruit retention, and marketable fruit and the yield, whereas reducing the irrigation level from 100%ETo to 60%ETo decreased fruit retention from 76.54% to 64.24% in 2018 and from 76.24% to 64.93% in 2019 (Table 5). These findings are in agreement with those obtained by Khattab et al. (2011) and will be useful for informing irrigation management to enhance Wonderful pomegranate production in regions with similar soils and climates. In field experiments on mature drip-irrigated pomegranate trees in Egypt during the 2016 and 2017 production seasons, Taha (2018) found that average fruit yields of 40.2, 38.6, 36.9, 23.8, and 31.8 t/ha were obtained from trees supplied with 13,520, 11,270, 9,020, 6,760, and 18,075 m^3^/ha irrigation water, which were equivalent to 120%, 100%, 80%, and 60% ETo and local farmer practice, respectively. However, the local farmer practice was done by applying irrigation and fertilizer quantities without consulting from specialist in agriculture guidance and irrigation started in the second week of February and stopped after harvesting in September and minimum quantities of irrigation water were applied during the rest of the growing season.

**Table 5:** Main effects of the three irrigation treatments on the percentages of fruit set, drop, retention, sunburn, and cracking, the percentage of marketable fruit, and the yield of pomegranate (Punica granatum “Wonderful”) in the 2018 and 2019 seasons. Mean values within a column for each season that are followed by different letters are significantly different at *P* ≤ 0.05.

Season	Irrigation treatment	Fruit set (%)	Fruit drop (%)	Fruit retention (%)	Fruit cracking (%)	Fruit sunburn (%)	Marketable fruit (%)	Yield (kg/tree or t/ha)
2018	I_1_	80.61b	23.46b	76.54	20.35a	7.60b	72.05b	51.32b
	I_2_	88.38a	22.35b	77.65a	7.66c	7.35b	84.99a	54.56a
	I_3_	70.95c	35.76a	64.24b	19.76b	18.01a	62.23c	41.84c
	LSD	1.24	1.36	1.36	0.24	0.3	0.44	0.19
2019	I_1_	80.60b	23.76b	76.24a	21.05a	7.13b	71.82b	50.98b
	I_2_	89.59a	23.33b	76.67a	7.27c	7.05b	85.69a	54.79a
	I_3_	69.26c	35.08a	64.93b	19.45b	19.23a	61.33c	41.08c
	LSD	1.46	1.36	1.36	0.19	0.26	0.31	0.18

In terms of the fertilizer regime, the application of lower amounts of mineral fertilizer in combination with organic manure (i.e., 50% mineral fertilizer + 50% organic manure or 25% mineral fertilizer + 75% organic manure) gave reasonable values for the percentages of fruit cracking, fruit set, fruit sunburn, fruit drop, fruit retention, and marketable fruit and the yield of Wonderful pomegranate trees in the 2018 and 2019 seasons compared with 100% mineral fertilizer or 100% organic manure (Table 6). The maximum percentages of fruit set (2018 and 2019: 86.40% and 85.91%, respectively) and fruit retention (77.27% and 77.37%, respectively) and the maximum yield (53.03 kg/tree and 52.72 kg/tree, respectively) were obtained under treatment fertilization regime of 75% mineral fertilizer + 25% organic manure , while the maximum percentage of marketable fruit (74.59% and 74.34%, respectively) was obtained under fertilization regime of 50% mineral fertilizer + 50% organic manure in both seasons (Table 6). These results can be attributed to the fact that organic manure helped to facilitate the availability and uptake of most nutrients to the trees, resulting in an increased fruit yield, and support reports by Rabeh, El-Koumey & Akasem (1993) and Huang, Zhang & Qian (1995) that treating Balady mandarin (*Citrus deliciosa* Ten.) and Satsuma mandarin (*Citrus unshiu* Marc.) trees with biofertilizers alone or in combination with organic manure stimulated plant root growth, nutrient absorption, and photosynthesis, leading to an increased yield. Similarly, in a field experiment conducted by Mansour (2018) on 8-year-old Wonderful pomegranate trees in Egypt during the 2015–2017 production seasons. The experiment comprised of two levels of humic acid (0 and 50 g/tree/season) and five nitrogen fertilizers form chicken manure, compost, cattle manure, mineral nitrogen as experiment control 40 kg actual N/fed (fed = 4,200 m^2^) and mineral nitrogen as orchard control 80 kg actual N/fed. He found that the trees could be fertilized at a rate of 40 kg N/fed/year instead of 80 kg N/fed/year and that the application of compost or chicken manure at a rate of 40 g N/tree/year with or without the addition of humic acid at a rate of 50 g/tree/year could improve the physical and chemical properties of the fruit and yield while decreasing environmental pollution.

**Table 6:** Main effects of the five fertilizer regimes on the percentages of fruit set, drop, retention, sunburn, and cracking, the percentage of marketable fruit, and the yield of pomegranate (Punica granatum “Wonderful”) in the 2018 and 2019 seasons. Mean values within a column for each season that are followed by different letters are significantly different at *P* ≤ 0.05.

Season	Fertilizer regime	Fruit set (%)	Fruit drop (%)	Fruit retention (%)	Fruit cracking (%)	Fruit sunburn (%)	Marketable fruit (%)	Yield (kg/tree or t/ha)
2018	T_1_	78.77c	26.71c	73.29c	19.64a	11.37c	69.00c	48.17c
	T_2_	72.26d	32.55a	67.45e	13.41e	13.17a	73.42b	46.32e
	T_3_	86.40a	22.73e	77.27a	17.02b	8.73e	74.25a	53.03a
	T_4_	83.23b	24.55d	75.45b	15.63c	9.78d	74.59a	50.84b
	T_5_	79.25c	29.42b	70.58d	13.94d	11.87b	74.19a	47.83d
	LSD	1.60	1.75	1.75	0.31	0.39	2.06	0.24
2019	T_1_	78.61c	26.73c	73.27b	18.86a	11.44c	69.69d	47.74c
	T_2_	72.64d	33.43a	66.57c	13.70e	13.77a	72.53c	46.08d
	T_3_	85.91a	22.63d	77.37a	17.13b	8.96e	73.91b	52.72a
	T_4_	82.44b	24.31d	75.69a	15.89c	9.77d	74.34a	50.50b
	T_5_	79.49c	29.84b	70.17c	14.02d	11.73b	74.25ba	47.70c
	LSD	1.88	1.75	1.75	0.25	0.33	0.40	0.23

### Effect of the interaction between the irrigation treatment and fertilizer regime on nutritional status

In both seasons, the percentage contents of N, K, P, Mg, and chlorophyll were highest when the 100% ETo irrigation treatment was combined with the fertilizer regime of 75% mineral fertilizer + 25% organic manure, with values of 1.99%, 0.61%, 1.72%, 0.51%, and 51.25 SPAD, respectively, for 2018 and 2.08%, 0.67%, 1.76%, 0.52%, and 54.33 SPAD, respectively, for 2019 (Table 7). By contrast, the Ca content was highest when 100% ETo was combined with fertilizer regime of 100% organic manure, which gave values of 0.99% in 2018 and 1.05% in 2019 (Table 7). Irrigation regime of 100% ETo in combination with fertilizer regime of 75% mineral fertilizer + 25% organic manure instead of 100% mineral fertilizer improved the concentration of N by 5.99%, P by 14.29%, K by 4.50%, Mg by 13.19%, and chlorophyll contents by 12.04% as a mean of the two growing seasons, while irrigation regime of 100% ETo combined with 100% organic manure instead of 100% mineral fertilizer increased the content of Ca by 20.71%.

**Table 7:** Interaction effect of the three irrigation treatments and five fertilizer regimes on the N, K, P, Ca, Mg, and chlorophyll contents of pomegranate (*Punica granatum* “Wonderful”) leaves in the 2018 and 2019 seasons.

Season	Treatment	N (%)	P (%)	K (%)	Ca (%)	Mg (%)	Chlorophyll (SPAD)
2018	I_1_+ T_1_	1.91	0.53	1.64	0.82	0.44	45.85
	I_1_+ T_2_	1.79	0.45	1.53	0.99	0.37	39.45
	I_1_+ T_3_	1.99	0.61	1.72	0.87	0.51	51.25
	I_1_+ T_4_	1.96	0.57	1.68	0.91	0.47	48.3
	I_1_+ T_5_	1.85	0.48	1.61	0.95	0.43	44.43
	I_2_+ T_1_	1.84	0.41	1.47	0.71	0.37	39.55
	I_2_+ T_2_	1.68	0.36	1.37	0.84	0.33	36.65
	I_2_+ T_3_	1.94	0.47	1.59	0.73	0.41	43.15
	I_2_+ T_4_	1.89	0.43	1.53	0.78	0.39	40.25
	I_2_+ T_5_	1.75	0.38	1.43	0.82	0.34	38.68
	I_3_+ T_1_	1.75	0.35	1.31	0.56	0.27	33.03
	I_3_+ T_2_	1.63	0.30	1.28	0.7	0.24	33.43
	I_3_+ T_3_	1.92	0.48	1.42	0.57	0.35	38.33
	I_3_+ T_4_	1.86	0.42	1.38	0.63	0.33	36.7
	I_3_+ T_5_	1.81	0.39	1.35	0.67	0.29	35.55
	LSD	0.02	0.0095	0.009	0.009	1.15	0.35
2019	I_1_+ T_1_	1.93	0.59	1.69	0.87	0.47	48.38
	I_1_+ T_2_	1.8	0.54	1.59	1.05	0.42	45.13
	I_1_+ T_3_	2.08	0.67	1.76	0.92	0.52	54.33
	I_1_+ T_4_	1.98	0.64	1.73	0.95	0.49	42.48
	I_1_+ T_5_	1.86	0.56	1.65	1	0.44	46.83
	I_2_+ T_1_	1.91	0.48	1.6	0.76	0.41	41.58
	I_2_+ T_2_	1.76	0.38	1.47	0.92	0.35	38.05
	I_2_+ T_3_	1.98	0.56	1.69	0.81	0.47	46.88
	I_2_+ T_4_	1.95	0.52	1.65	0.85	0.44	43.03
	I_2_+ T_5_	1.84	0.43	1.56	0.88	0.38	39.43
	I_3_+ T_1_	1.67	0.32	1.27	0.59	0.25	32.65
	I_3_+ T_2_	1.51	0.27	1.25	0.73	0.23	31.95
	I_3_+ T_3_	1.81	0.43	1.38	0.61	0.33	37.93
	I_3_+ T_4_	1.76	0.39	1.35	0.66	0.31	35.9
	I_3_+ T_5_	1.72	0.36	1.32	0.7	0.26	34.73
	LSD	0.02	0.01	0.01	0.01	0.008	4.66

### Effect of the interaction between the irrigation and fertilizer regimes on fruit characteristics and yield

All of the fruit characteristics measured were significantly affected by the interaction between the irrigation treatment and fertilizer regime (Table 8). The lowest levels of fruit cracking, fruit sunburn, and fruit drop observed were 7.01%, 5.32%, and 15.87%, respectively, in 2018 and 7.15%, 5.18%, and 16.81%, respectively, in 2019. Decreasing the amount of irrigation water to 60% ETo and adding 100% organic manure to each tree reduced the percentage of fruit cracking by 28.67% as an average of the two growing seasons compared with the control treatment (100% ETo and 100% mineral fertilizer), while decreasing the amount of irrigation water to 80% ETo and adding 75% mineral fertilizer + 25% organic manure to each tree reduced the percentage of fruit sunburn by 26.52%, and decreasing the amount of irrigation water to 60% ETo and adding 100% mineral fertilizer to each tree decreased the percentage of fruit drop by 8.13% compared with the control. The other treatments gave intermediate values.

**Table 8:** Interaction effect of the irrigation and fertilizer regimes on the percentages of fruit set, fruit cracking, fruit drop, fruit retention, fruit sunburn, marketable fruit and the yield of pomegranate (*Punica granatum* “Wonderful”) in the 2018 and 2019 seasons.

Season	Treatment	Fruit set (%)	Fruit drop (%)	Fruit retention (%)	Fruit cracking (%)	Fruit sunburn (%)	Marketable fruit (%)	Yield (kg/tree or t/ha)
2018	I_1_+ T_1_	80.61	21.93	78.07	22.83	7.36	69.81	50.75
	I_1_+ T_2_	71.91	31.59	68.41	17.82	9.24	72.94	48.75
	I_1_+ T_3_	87.75	19.37	80.63	22.22	5.77	72.01	54.62
	I_1_+ T_4_	84.31	20.89	79.11	20.73	6.76	72.5	52.74
	I_1_+ T_5_	78.46	23.53	76.47	18.17	8.84	72.99	49.72
	I_2_+ T_1_	87.75	21.35	78.65	9.17	6.8	84.02	54.16
	I_2_+ T_2_	80.58	26.98	73.02	5.99	9.29	84.72	50.68
	I_2_+ T_3_	94.42	15.87	84.13	8.68	5.32	86.00	59.57
	I_2_+ T_4_	91.53	18.91	81.09	7.46	6.31	86.23	56.46
	I_2_+ T_5_	87.64	28.64	71.36	7.01	8.99	83.99	51.91
	I_3_+ T_1_	67.95	36.83	63.17	26.9	19.93	53.17	39.59
	I_3_+ T_2_	64.29	39.09	60.91	16.42	20.99	62.59	39.52
	I_3_+ T_3_	77.04	32.94	67.06	20.16	15.08	64.76	44.89
	I_3_+ T_4_	73.84	33.85	66.15	18.7	16.26	65.04	43.32
	I_3_+ T_5_	71.64	36.09	63.91	16.64	17.78	65.58	41.88
	LSD	2.18	2.18	2.18	0.35	0.43	0.58	0.26
2019	I_1_+ T_1_	80.61	22.05	77.95	23.6	6.93	69.47	50.47
	I_1_+ T_2_	71.91	31.82	68.18	18.55	8.94	72.51	48.74
	I_1_+ T_3_	86.75	18.91	81.09	22.93	5.51	71.55	53.83
	I_1_+ T_4_	83.31	20.34	79.66	21.45	6.21	72.34	52.22
	I_1_+ T_5_	80.42	25.67	74.33	18.72	8.05	73.23	49.63
	I_2_+ T_1_	88.79	22.15	77.85	8.71	6.35	84.93	53.78
	I_2_+ T_2_	82.43	29.07	70.93	5.86	9.35	84.79	50.84
	I_2_+ T_3_	95.55	16.81	83.19	8.11	5.18	86.71	60.10
	I_2_+ T_4_	92.57	19.62	80.38	7.15	6.01	86.84	56.92
	I_2_+ T_5_	88.6	29.01	70.99	6.52	8.33	85.15	52.31
	I_3_+ T_1_	66.41	36	64	24.28	21.05	54.67	38.99
	I_3_+ T_2_	63.58	39.41	60.59	16.7	23.01	60.29	38.67
	I_3_+ T_3_	75.43	32.17	67.83	20.35	16.18	63.46	44.23
	I_3_+ T_4_	71.42	32.97	67.03	19.07	17.08	63.85	42.36
	I_3_+ T_5_	69.43	34.82	65.18	16.83	18.81	64.35	41.17
	LSD	2.05	2.12	2.12	0.38	0.43	0.56	0.31

Reduced percentages of fruit cracking, fruit sunburn, and fruit drop were observed under deficit irrigation when both mineral and organic fertilizers were used. By contrast, in a study on pomegranate trees of the Manfalouty cultivar in 2007 and 2008, Khattab et al. (2011) found that irrigation at a rate of 15 m^3^/tree/year gave the lowest fruit drop percentages (8.75% and 9.09%, respectively), while 7 m^3^/tree/year gave the highest fruit drop percentages (17.15% and 17.88%, respectively) in both seasons, and that 13 m^3^/tree/year gave the lowest fruit cracking percentage (6.02% and 6.09%, respectively) followed by 11 m^3^/tree/year (6.85% and 6.72%, respectively) and 9 m^3^/tree/year (7.27% and 8.22%, respectively) in both seasons. The reduction in fruit cracking, fruit sunburn, and fruit drop with a decreasing amount of irrigation water observed in the present study may be attributed to the pomegranate fruit being taken from trees that were growing under no water stress due to the presence of organic manure, whereas Khattab et al. (2011) illustrated that a decrease in applied irrigation water increases fruit cracking when fruit are taken from trees growing under water stress.

By exploring the data presented in Table 8, it is clear that all of the parameters that describe the characteristics of the studied fruit were significantly affected by the interaction between the irrigation regime and fertilizer regime. The percentages of fruit set, fruit retention, and marketable fruit and the yield reached maximum values of 94.42%, 84.13%, 86.23%, and 59.57 kg/tree, respectively, in 2018 and 95.55%, 83.19%, 86.84%, and 60.10 kg/tree, respectively, in 2019. Decreasing the amount of irrigation water to 80% ETo and applying 75% mineral fertilizer + 25% organic manure increased the yield by 18.23% as an average of the two growing seasons compared with the control (integrated 100% ETo and 100% mineral fertilizer), while decreasing the amount of irrigation water to 80% ETo and adding 100% organic manure increased the percentage of marketable fruit by 21.82%, decreasing the amount of irrigation water to 80% ETo and adding 25% mineral fertilizer + 75% organic manure increased the percentage of fruit set by 17.83%, and combining 100% ETo with 75% mineral fertilizer + 25% organic manure improved the percentage of fruit retention by 3.65% compared with the control. The finding that deficit irrigation in combination with organic manure with mineral fertilizer or organic manure alone gave the highest yield and percentage of marketable fruit may be attributed to the fact that the presence of organic manure reduces water stress for the growing plants and indicates that the yield of Wonderful pomegranate was not sensitive to water stress under the experimental conditions when organic manure fertilizer was added. Similarly, Intrigliolo et al. (2013) suggested that a mild deficit of water during the flowering and fruit set periods, which saved 9%–14% water, was the best strategy for cultivating Mollar de Elche pomegranate trees, as this resulted in minimal negative effects on fruit yield.

### Irrigation water use efficiency

**Figure 3:**
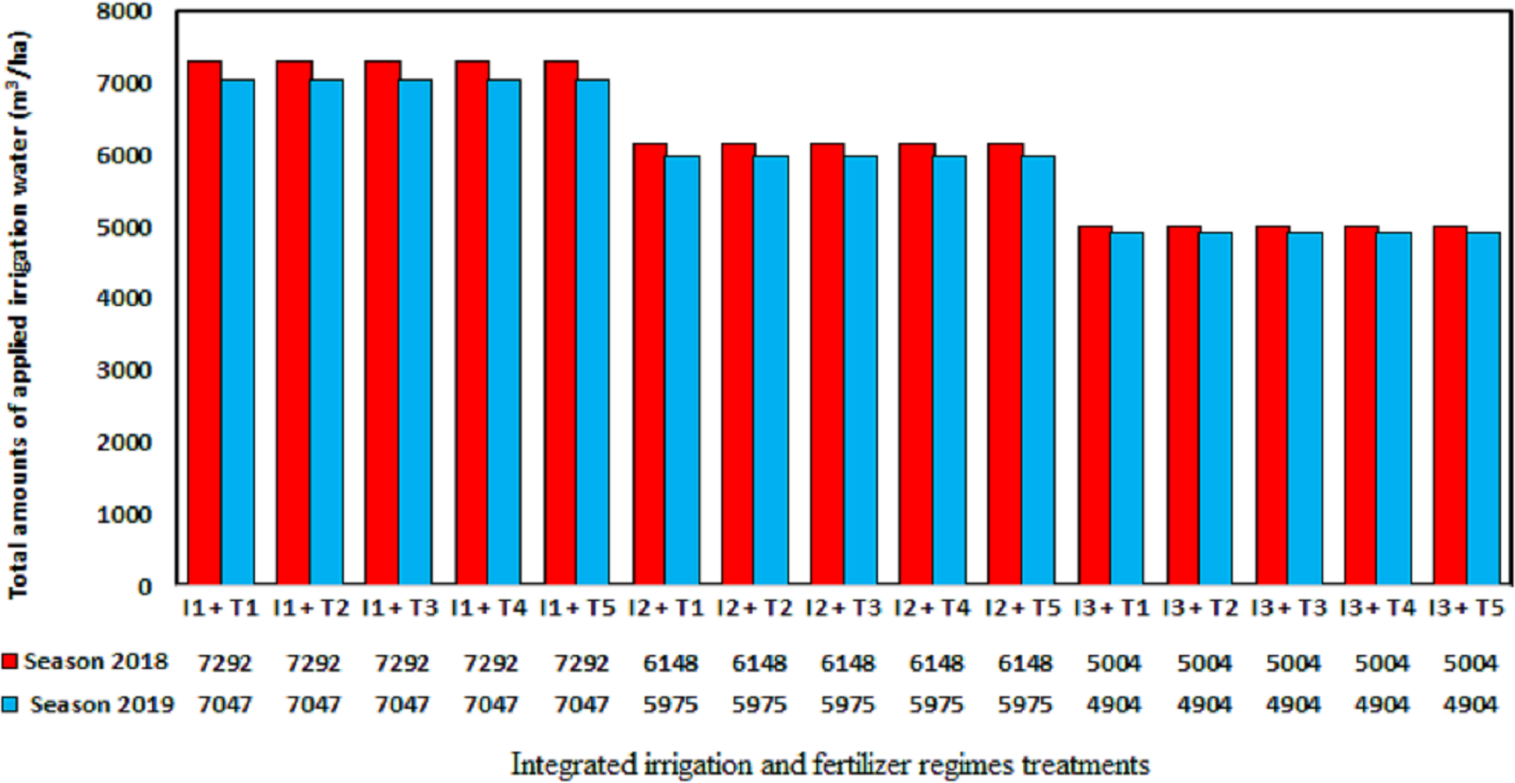
Seasonal irrigation applied water including the effective rain were as affected by integrated irrigation and fertilizer regimes treatments.

**Figure 4:**
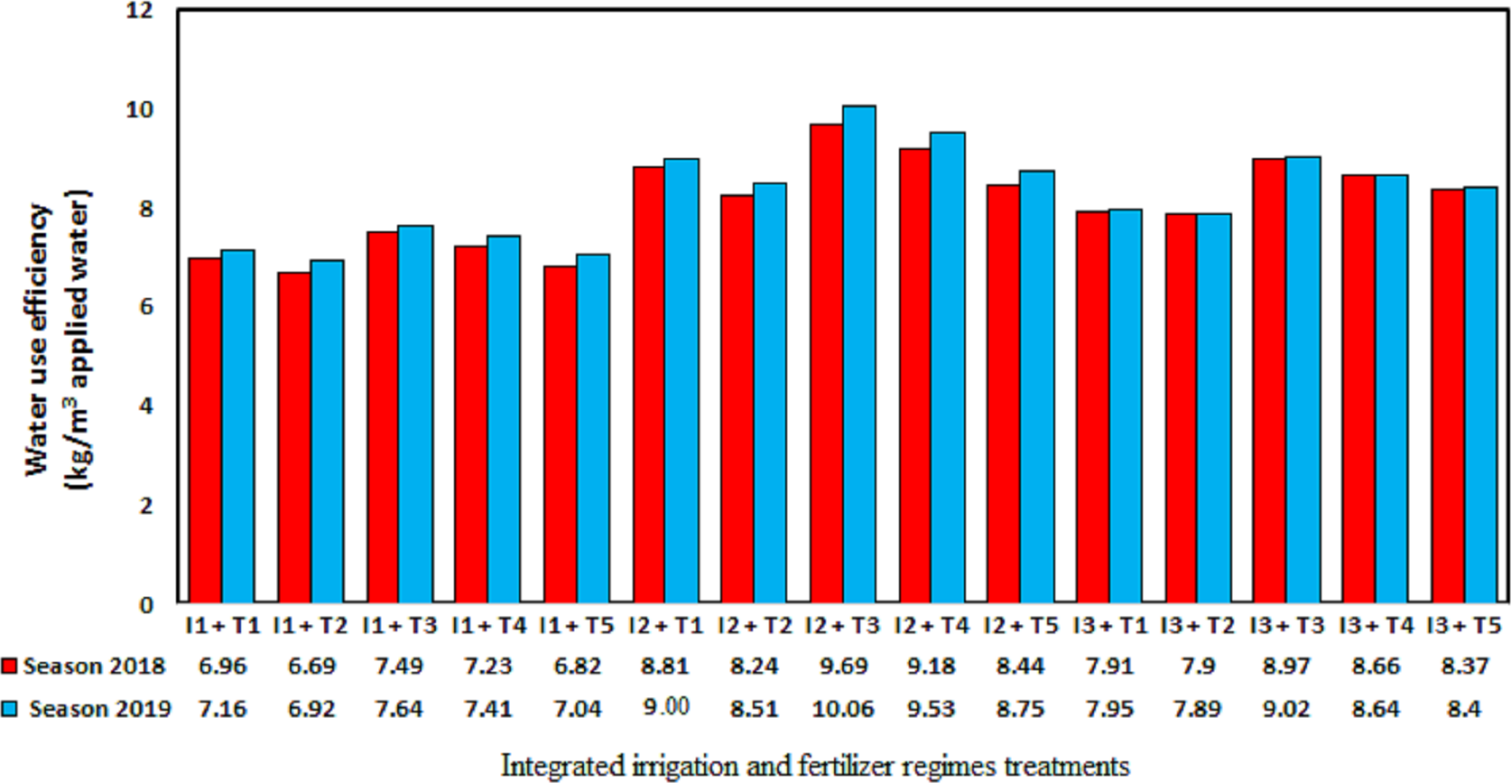
Seasonal irrigation applied water including the effective rain were as affected by integrated irrigation and fertilizer regimes treatments.

The irrigation water use efficiency index can support decision making around on-farm irrigation (Fernández et al., 2020). In this study, the irrigation water use efficiency was defined as the yield (kg/ha) divided by the amount of irrigation water applied (crop evapotranspiration; m^3^/ha plus the effective rain). By contrast, the irrigation water productivity is defined as the yield (kg/ha) divided by the total amount of water applied by irrigation (irrigation applied + rainfall; m^3^/ha) (Volschenk, 2020). Seasonal irrigation applied water including the effective rain were depicted in Fig. 3 as affected by integrated irrigation and fertilizer regimes. However, the total amounts of applied irrigation water were 7,292, 6,148, and 5,004 m^3^/ha with the 100% ETo, 80% ETo, and 60% ETo treatments, respectively, in 2018 and 7,047, 5,975, and 4,904 m^3^/ha, respectively, in 2019. The maximum water use efficiency (Fig. 4) was obtained with the 80% ETo treatment combined with 75% mineral fertilizer + 25% organic manure in both seasons with values of 9.69 and 10.06 kg/m^3^ applied water, respectively. By contrast, Martínez-Nicolás et al. (2019) demonstrated that withholding irrigation during the flowering and fruit set periods for the pomegranate cultivars Mollar de Elche and Wonderful gave irrigation water productivities of 5.6 and 7.1 kg/m^3^, respectively, whereas Parvizi, Sepaskhah & Ahmadi (2014) found that deficit irrigation applied at 75% and 50% ETc increased the irrigation water productivity of the cultivar Rabab on average over two seasons compared with trees that were irrigated at 100% ETc (irrigation water productivity, 4.2 kg/m^3^).

The application of 60% ETo in combination with 75% mineral fertilizer + 25% organic manure gave yields of 44.89 and 44.23 t/ha in 2018 and 2019, respectively, giving irrigation water use efficiencies of 8.97 and 9.02 kg/m^3^ applied water, respectively. Thus, this irrigation and fertilizer regime can be recommended in situations where water is scarce or where production is focused on the delivery of pomegranate fruit to the industry (Intrigliolo et al., 2012). The other treatments provided intermediate values of irrigation water use efficiency, although deficit irrigation is expected to increase water use efficiency due to an increase in irrigation efficiency, as described by Intrigliolo et al. (2012) and Tavousi et al. (2015).

## Conclusions

The findings of this study indicate that the application of mineral fertilizer and organic manure in combination with different levels of water irrigation can improve the growth of Wonderful pomegranate trees and produce yields that match those of existing production practices. Furthermore, deficit irrigation had a positive effect on the irrigation water use efficiency despite the sensitivity of pomegranate production to water supply. Consequently, a combination of 80% ETo and 75% mineral fertilizer + 25% organic manure is recommended in those cases where the price of irrigation (water + energy) is high or where production is focused on the delivery of pomegranate fruit to the industry.

##  Supplemental Information

10.7717/peerj.10979/supp-1 Data S1 Primary data

## Additional Information and Declarations

### Data Availability

The following information was supplied regarding data availability: Raw measurements are available as a Supplemental File.
